# Syphilis and Epilepsy

**Published:** 1910-05-21

**Authors:** 


### Medicine.

### SYPHILIS AND EPILEPSY.

There is evidence, based upon statistics collected both in Paris and in America independently, that from 10 to 16 per cent. of epileptics have had syphilis, and it appears that epilepsy may occur either in the secondary stage of syphilis or in the tertiary stage. When tertiary, epilepsy may be the first sign that anything is wrong, or it may occur consecutive to or in the midst of other symptoms of syphilis, especially in association with frequent or intense pains in the head, either localised or general, with sleepiness, lassitude, and muscular fatigue. Where the line is to be drawn between what is generally regarded as cerebral syphilis upon the one hand and syphilitic epilepsy upon the other is very difficult to say; but it is certain that convulsions do occur, not only in the form of Jacksonian epilepsy, such as might be expected from irritation of the motor cortex by a gumma or by chronic thickening of the dura mater, but also as true epileptic attacks which in themselves are precisely similar to epilepsy which is due to alcohol, heredity, or other causes. In syphilitic cases it is frequently not more than part of a group of symptoms which may include almost any that cerebral disorganisation may give rise to, particularly heaviness of the head, cephalalgia, vertigo, mental confusion, numbness and tingling of the limbs, amblyopia, buzzing in the ears, deafness, apathy, diminished aptitude for mental work, partial and incomplete paralysis, threatenings of hemiplegia, followed by true paralysis, ocular palsies, monoplegias, and hemiplegia.

### Prognosis.

The prognosis depends upon the state of the disease, upon the accompanying symptoms, and upon the character of the lesions, which may be either capable of resolution, as in the case of a gumma, or incompatible of resolution when actual cerebral softening has taken place. The fewer the symptoms of cerebral disease the better the prognosis; the severity of the individual epileptic seizures does not necessarily go parallel with the prognosis, provided several attacks of epilepsy are not associated with grave concomitant symptoms. Epilepsy arising during the secondary stage of syphilis may vanish completely under treatment, while tertiary epilepsy is far less hopeful.

